# Drug-induced adverse events in modern pharmacotherapy: mechanisms, clinical manifestations, and implications for risk assessment and management

**DOI:** 10.3389/fphar.2026.1857708

**Published:** 2026-07-10

**Authors:** Zhangyurong Chen, Xinrui Zhong, Mengying Jiang, Weiyue Liu, Kaicong Cai

**Affiliations:** 1 College of Chemistry and Materials Science, Fujian Provincial Key Laboratory of Advanced Materials Oriented Chemical Engineering, Fujian Normal University, Fuzhou, China; 2 Fujian Provincial Key Laboratory of Theoretical and Computational Chemistry, Xiamen University, Xiamen, China; 3 Fujian Provincial Key Laboratory of Featured Biochemical and Chemical Materials, Ningde Normal University, Ningde, China

**Keywords:** drug safety, drug-induced adverse events, real-world evidence, risk stratification, toxicity mechanisms

## Abstract

Drug-induced adverse events remain a major challenge in modern pharmacotherapy, particularly with the increasing use of targeted therapies, biologics, and combination regimens. In clinical practice, toxicity is often difficult to predict, interpret, and manage because adverse events arise from complex interactions between drug mechanisms, patient susceptibility, and treatment context. This review provides a clinically oriented framework for understanding drug-induced adverse events across multiple levels. We first summarize key mechanistic drivers, including pathway perturbation, off-target effects, metabolic and mitochondrial dysfunction, and immune dysregulation. We then discuss how these mechanisms translate into organ-specific toxicity patterns involving the liver, heart, kidney, nervous system, and immune system, which represent the most common clinical presentations. In addition, we examine how safety profiles are defined and refined through different layers of evidence, including randomized clinical trials, meta-analyses, and real-world pharmacovigilance data. These complementary evidence sources are essential for identifying both common and rare adverse events, particularly those that emerge after broader clinical use. Importantly, this review highlights practical considerations for clinical risk assessment and management. We discuss key factors influencing toxicity risk, including patient comorbidity, polypharmacy, and baseline organ function, as well as the role of dynamic monitoring, biomarkers, and early signal detection. Emphasis is placed on translating mechanistic insight into actionable strategies for prevention, early recognition, and individualized management of adverse events. Overall, drug safety should be viewed as a dynamic and context-dependent process. Integrating mechanistic understanding with clinical evidence and real-world data can improve risk prediction and support more effective and personalized pharmacotherapy.

## 1 Introduction

Modern pharmacotherapy is increasingly defined by molecularly targeted agents, immune-modulating therapies, biologics, complex combination regimens, long-term maintenance strategies, and treatment in patients with multiple comorbidities. These changes have made adverse-event profiles more heterogeneous, mechanism-dependent, and context-sensitive than those traditionally captured by simple organ-based toxicity lists. A contemporary safety framework therefore needs to integrate mechanistic toxicology, clinical evidence, real-world surveillance, and predictive toxicology perspectives to explain not only what adverse events occur, but why they occur, in whom they are most likely to occur, and how they can be anticipated or mitigated in practice (U.S. Food and Drug Administration, 2009; International Council for Harmonisation, 2004; U.S. Food and Drug Administration, 2024).

Drug-induced adverse events have become increasingly complex in modern pharmacotherapy, particularly with the expansion of targeted therapies, biologics, and combination regimens. In contrast to traditional small-molecule treatments, safety profiles are often delayed, population-dependent, and difficult to fully characterize within controlled clinical trials. Evidence from real-world pharmacovigilance and post-marketing analyses indicates that clinically relevant toxicities frequently emerge only after broader clinical use, especially under conditions of long-term exposure or therapeutic combination (Yin et al., 2022; Chen J. et al., 2025; Liu H. et al., 2025).

These observations challenge the conventional view of safety as a fixed property defined at the time of drug approval. Instead, adverse events are better understood as context-dependent outcomes shaped by treatment duration, patient heterogeneity, and biological system interactions (Chen J. et al., 2024; Chen B. et al., 2024; Zeng et al., 2025). Importantly, many toxicities are not adequately explained as nonspecific off-target effects alone, but instead reflect biologically consequential pathway perturbation expressed differently across tissues, treatment settings, and physiological conditions (Liu L. et al., 2024; Ren S. et al., 2024; Wei et al., 2024). This overlap makes it difficult to interpret toxicity within a purely target-centric framework.

At the same time, no single source of evidence is sufficient to define drug safety. Systematic reviews, network meta-analyses, and pharmacovigilance studies each capture different dimensions of risk, but are often interpreted in isolation (Feng Y. et al., 2023; Xie et al., 2023; Jiao et al., 2024; Wang P. et al., 2024). This fragmentation limits the ability to form coherent and clinically actionable safety assessments. Integrating mechanistic understanding with clinical and population-level evidence therefore remains a central challenge in contemporary pharmacotherapy (Pan et al., 2023; Tian et al., 2023b; Xiao et al., 2023).

**FIGURE 1:**
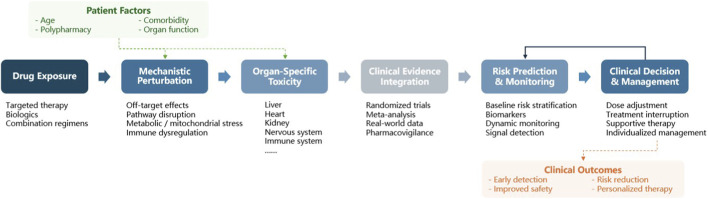
Translational framework for drug-induced adverse events in modern pharmacotherapy.

In this review, we propose a translational framework for understanding drug-induced adverse events that links mechanistic toxicity, organ-specific manifestations, evidence synthesis, and risk management. The overall conceptual structure of this framework is summarized in Figure 1. We first examine pathway-related and off-target mechanisms of toxicity, followed by metabolic and immune-mediated injury. We then discuss how these processes manifest at the organ level and how safety profiles are shaped through clinical trials and real-world data. Finally, we address emerging strategies for risk prediction and management, including approaches informed by large-scale pharmacovigilance data and artificial intelligence (Mao et al., 2024; Zhang M. et al., 2025). This review also aims to provide clinically actionable insights to support risk assessment and management in routine practice.

**FIGURE 2:**
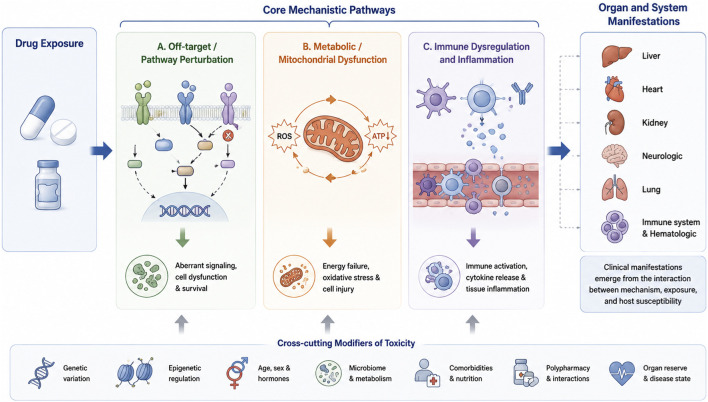
Mechanistic landscape of drug-induced toxicity (Major pathways and cellular perturbations linking drug exposure to organ-specific toxicities).

Drug exposure can lead to adverse events through interacting mechanistic pathways, including off-target or pathway perturbation, metabolic and mitochondrial dysfunction, and immune dysregulation or inflammation (Figure 2). These mechanisms converge with host modifiers such as genetic variation, epigenetic regulation, age, sex, microbiome, comorbidity, polypharmacy, organ reserve, and disease state to produce organ- and system-specific manifestations.

**FIGURE 3:**
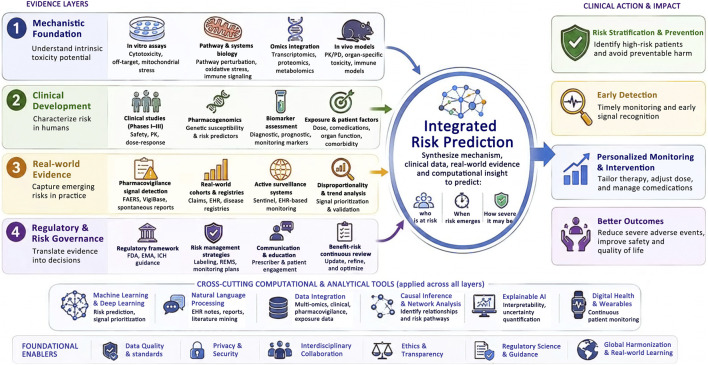
Predictive toxicology framework linking mechanistic evidence, clinical development, real-world evidence, regulatory risk governance, and computational tools to clinical action. Multi-layer evidence from *in vitro* and *in vivo* models, pharmacogenomics, biomarker assessment, pharmacovigilance, active surveillance, and regulatory review can be integrated to support risk stratification, early detection, personalized monitoring, and improved patient outcomes.

## 2 Off-target and pathway-related toxicity

Toxicity is often hardest to interpret when the intended pharmacologic target is not the only biology being perturbed. In that setting, adverse events emerge through pathway spillover, compensatory rewiring, and tissue-specific vulnerability rather than through a simple failure of selectivity (Querfurth et al., 2022; Zhang C. et al., 2024; Kciuk et al., 2025; Hushmandi et al., 2026).

One persistent challenge in modern pharmacotherapy is that molecular precision does not eliminate systems-level toxicity (Pang et al., 2023; Li X. et al., 2025). Even highly selective agents can trigger adverse reactions when signaling nodes essential for disease control in one compartment remain physiologically important in another. The resulting injuries may involve vascular homeostasis, immune regulation, electrophysiology, coagulation, mitochondrial signaling, or tissue repair, and they often become visible only when treatment moves beyond narrowly defined trial populations (Wang Z. et al., 2023; Wei Q. et al., 2023).

Across targeted therapies, pathway-related toxicity often represents on-target pharmacology expressed in the wrong tissue, at the wrong intensity, or under the wrong combinatorial conditions (Di et al., 2022; Wang Y.-J. et al., 2022; Xing et al., 2023). Kinase inhibitors, particularly EGFR- and JAK-targeting agents, provide a representative model, because suppression of one signaling cascade can alter compensatory networks involving PI3K/AKT, MAPK, and JAK/STAT pathways, thereby generating hypertension, rash, myelosuppression, mucosal injury, or inflammatory complications (Duan et al., 2022; Wei Q. et al., 2023). Similar logic applies to biologics and antibody-drug conjugates, where disruption of a therapeutically desirable axis may destabilize regulatory circuits and expose latent toxic phenotypes once treatment is extended to broader populations (Yang et al., 2018; Wei Y. et al., 2023). Toxicity is therefore better understood as a systems-level consequence of pathway redistribution rather than as a simple appendage to efficacy.

Evidence synthesis across targeted therapies indicates that the same drug class can produce superficially similar safety signals through distinct pathway architectures (Jiang et al., 2024b; Cai W. et al., 2025). Some adverse events arise from direct inhibition of the intended target in normal tissues, whereas others reflect indirect pathway crosstalk, altered cytokine signaling, or downstream metabolic compensation (Zhang Y. et al., 2022; He et al., 2023). Cross-trial comparisons and mechanistic analyses are therefore essential for distinguishing class-defining toxicities from molecule-specific or schedule-dependent effects (Yuan et al., 2022; Chen X. et al., 2025). This supports a more useful framework for interpretation: direct target extension, network spillover, compensatory activation, and pharmacodynamic overreach (He Hailun et al., 2021; Liao et al., 2021; Zhang et al., 2021).

The core literature further suggests that pathway-related toxicities become particularly significant when therapies are used in combinations that collapse multiple compensatory systems simultaneously (Tong et al., 2021; Wang et al., 2021; Yu et al., 2021; Ding et al., 2022b). Combination regimens may improve disease control, but they also increase the probability that overlapping signaling stress will amplify endothelial dysfunction, immune activation, electrophysiologic instability, or impaired tissue repair (Tong et al., 2021; Duan et al., 2022). In this context, toxicity is not merely additive but emergent (Liu et al., 2022). Comparative studies and meta-analyses help identify whether toxicity burden rises with pathway depth and exposure duration (Jiang et al., 2024b; Li and Xu, 2025).

Supportive studies broaden this picture by showing how off-target toxicity is shaped by patient context rather than drug properties alone (Liu et al., 2024c; Hu et al., 2025). Age, comorbidity, polypharmacy, and inflammatory status can lower the threshold at which pathway perturbation becomes symptomatic (Ma H. et al., 2022; Hao et al., 2024). Pharmacokinetic variability and exposure differences further influence clinical stability (Rauf et al., 2021; Zeng M. et al., 2024).

Across this literature, recurring patterns are easier to identify when pathway class, toxicity phenotype, and management implications are considered together (Chen et al., 2020a; Cui Ya et al., 2020; Xiong et al., 2021). Similar comparisons emerge when monotherapy is contrasted with combination regimens (Cao et al., 2020; Chen Y. et al., 2020; Li Y. et al., 2020). The temporal dimension also matters, as early biomarker changes may precede clinically significant toxicity (Ahmad et al., 2018; Li et al., 2019; Wu et al., 2019). Read together, these studies indicate that off-target injury is best understood as a transfer of pharmacologic stress across interconnected biologic systems rather than as a collection of isolated drug-specific complications. Representative examples of pathway-targeted therapies and their associated off-target toxicity mechanisms are summarized in Table 1.

**TABLE 1:** Representative examples of pathway-targeted therapies and associated off-target toxicities.

Drug Or Class With Examples	Target Or Pathway	Clinically Recognized Toxicity	Mechanistic Basis	Predictive Or Monitoring Implication	Candidate References
EGFR inhibitors (cetuximab, erlotinib, gefitinib, osimertinib)	EGFR signaling in epithelial and tumor cells	Acneiform or papulopustular rash, xerosis, paronychia, diarrhea	EGFR inhibition in skin and gastrointestinal epithelium impairs epithelial proliferation, barrier repair, and inflammatory homeostasis	Baseline dermatologic risk assessment, early rash grading, proactive skin management; rash can also indicate exposure or pharmacodynamic effect	(Lacouture, 2015): Fabbrocini 2015 EGFR inhibitor acneiform rash review; optional EGFR rash management guidance (Lacouture, 2015)
VEGF/VEGFR inhibitors (bevacizumab, sunitinib, sorafenib, lenvatinib)	VEGF ligand or VEGFR signaling	Hypertension, proteinuria, arterial thromboembolism, bleeding, impaired wound healing	Loss of VEGF-mediated endothelial nitric oxide signaling and endothelial repair increases vascular tone and microvascular injury	Blood pressure and urine protein monitoring; cardiovascular risk stratification before and during treatment	Bevacizumab hypertension literature; FDA label/guidance sources (Li and Kroetz, 2018)
JAK inhibitors (tofacitinib, baricitinib, upadacitinib)	JAK-STAT cytokine signaling	Serious infection, herpes zoster, malignancy, major adverse cardiovascular events, thrombosis	Broad cytokine pathway suppression alters host defense and inflammatory balance; risk is modified by age, cardiovascular risk, smoking, and immunosuppression	Patient selection, infection screening, vaccination, cardiovascular and thrombotic risk evaluation; boxed-warning informed prescribing	FDA 2021 JAK inhibitor safety communication; ORAL Surveillance (U.S. Food and Drug Administration, 2021)
PARP inhibitors (olaparib, niraparib, rucaparib)	PARP-mediated DNA repair	Anemia, thrombocytopenia, neutropenia, rare MDS/AML	Synthetic-lethality strategy also affects DNA repair capacity in hematopoietic progenitors	Complete blood count monitoring and dose interruption or reduction algorithms	PARP inhibitor labels; oncology safety reviews (Mizuno et al., 2025)
BCR-ABL and multikinase TKIs (ponatinib, nilotinib)	BCR-ABL plus off-target kinase networks	Arterial occlusive events, hypertension, metabolic vascular risk	Kinase selectivity profile intersects with endothelial, platelet, and metabolic pathways	Baseline cardiovascular risk assessment, lipid/glucose/BP monitoring, risk-adapted TKI selection	Cardio-oncology guidance; TKI cardiovascular safety studies (Lyon, 2022)
Immune checkpoint inhibitors (nivolumab, pembrolizumab, ipilimumab)	PD-1/PD-L1 or CTLA-4 immune checkpoints	Colitis, pneumonitis, endocrinopathies, myocarditis	Immune disinhibition produces tissue-specific autoimmunity; combination therapy increases risk	Baseline autoimmune/cardiac risk review; symptom-triggered workup; steroid and interruption algorithms	Salem 2018; Nguyen 2022; oncology irAE guidelines (Salem et al., 2018; Nguyen, 2022)
CAR-T and immune effector cell therapies (tisagenlecleucel, axicabtagene ciloleucel)	Engineered T-cell activation and cytokine signaling	Cytokine release syndrome and immune effector cell-associated neurotoxicity syndrome	Rapid immune-cell activation causes supraphysiologic cytokine release, endothelial activation, capillary leak, fever, hypotension, hypoxia, and neurotoxicity	ASTCT/CTCAE grading, early fever and hemodynamic monitoring, tocilizumab and corticosteroid algorithms	ASTCT CRS/ICANS consensus grading; CAR-T safety guidance (Lee, 2019)
Anti-TNF biologics (infliximab, adalimumab, etanercept)	TNF-alpha-mediated inflammatory signaling	Serious infection, tuberculosis reactivation, opportunistic infection, hepatitis B reactivation	TNF-alpha inhibition impairs granuloma maintenance, macrophage activation, and intracellular pathogen control	Latent TB/HBV screening, vaccination review, infection-risk stratification before treatment	Anti-TNF infection risk reviews; tuberculosis screening guidance (Ali et al., 2013)
SGLT2 inhibitors (canagliflozin, dapagliflozin, empagliflozin)	Renal glucose reabsorption via SGLT2	Euglycemic diabetic ketoacidosis, genital/urinary infections, volume depletion	Glycosuria and altered insulin-glucagon balance promote ketogenesis under fasting, acute illness, insulin deficiency, surgery, or low-carbohydrate intake	Sick-day rules, perioperative interruption, ketone assessment in symptomatic patients even with modest glucose elevation	FDA SGLT2 ketoacidosis warning; FAERS/clinical safety analyses (U.S. Food and Drug Administration, 2015)
GLP-1 receptor agonists (liraglutide, semaglutide, dulaglutide, exenatide)	GLP-1 receptor-mediated incretin and gastrointestinal motility pathways	Gastrointestinal intolerance, gallbladder/biliary disease signal, rare pancreatitis concern	Slowed gastric emptying, weight-loss-associated biliary changes, and altered gallbladder motility may contribute to selected events	Dose escalation, symptom-based biliary/pancreatic evaluation, risk-benefit framing in obesity/diabetes populations	JAMA Internal Medicine 2022 GLP-1RA gallbladder/biliary meta-analysis; drug labels (He et al., 2022)

Overall, off-target and pathway-related toxicity should increasingly be treated as a problem of mechanistic prediction (Figure 3) rather than retrospective description (Yu et al., 2021; Pang et al., 2023). The literature is moving toward integrated models that combine target biology, network topology, and early clinical signals (Cui et al., 2025). This perspective links pharmacology with precision safety and supports biomarker-informed risk mitigation strategies (Di et al., 2022; Wei Q. et al., 2023).

## 3 Metabolic and mitochondrial toxicity

Mechanistic diversity in drug toxicity often converges on a narrower set of metabolic vulnerabilities. Disturbances in cellular energetics and mitochondrial homeostasis help explain why clinically different adverse reactions can still share a common biologic core (Capela and Carvalho, 2022; Ding et al., 2022a; Buzibye et al., 2025; Turkington et al., 2025).

Many toxic reactions that appear clinically unrelated are linked by changes in bioenergetic resilience, redox balance, lipid handling, autophagy, and stress adaptation (Chen J. et al., 2022; Xie et al., 2022; Feng et al., 2024; Liu S. et al., 2024; Shen et al., 2024; Xiao and Chen, 2024). These effects are not always part of the intended treatment mechanism, yet they frequently shape tolerability once exposure is prolonged or combined with pre-existing physiologic strain. Adverse reactions may therefore present not as abrupt receptor-mediated events, but as slower disruptions in substrate utilization, mitochondrial dynamics, or oxidative signaling that eventually manifest as fatigue, organ dysfunction, steatosis, myopathy, neurotoxicity, or heightened inflammatory susceptibility (Chen J. et al., 2021; Raghu et al., 2021; Wang D. et al., 2023; Ma et al., 2024).

Mitochondria are not merely organelles injured by toxic drugs; they are integrative hubs that translate pharmacologic exposure into systemic vulnerability (Yuan et al., 2019; Cai et al., 2021). When mitochondrial respiration, membrane integrity, mitophagy, calcium handling, or reactive oxygen species buffering are disrupted, the downstream consequence is rarely restricted to one tissue type (Guo Y. et al., 2025). Instead, the phenotype depends on the organ’s energetic demand, regenerative capacity, and inflammatory background, which is why similar upstream insults can manifest as hepatotoxicity, cardiotoxicity, neurobehavioral change, renal injury, or impaired recovery after physiologic stress (Zhang P. et al., 2025).

Across therapeutic classes, metabolic toxicity is most convincing when it is read through a small set of recurring mechanistic motifs (Xiao et al., 2025; Zhu et al., 2025). One involves direct mitochondrial dysfunction, including impaired oxidative phosphorylation, disrupted electron transport, and altered ATP production (Bao et al., 2023; Shah et al., 2024). A second involves oxidative and redox stress, where excess reactive oxygen species or depleted antioxidant capacity produces cumulative cellular injury (Dong et al., 2023; Jiang et al., 2024c). A third involves disturbed lipid and glucose metabolism, often visible through steatosis, insulin signaling imbalance, altered fatty acid oxidation, or maladaptive substrate switching (Gao et al., 2023; Xiao et al., 2025). These motifs frequently overlap, and many agents do not activate a single pathway in isolation; rather, they create a metabolic cascade in which mitochondrial stress, inflammatory signaling, and organ-specific dysfunction amplify one another (Li Q. et al., 2023; Yin et al., 2023).

Recent studies also show that metabolic and mitochondrial toxicity often intersects with regulated cell death pathways such as apoptosis, ferroptosis, and defective autophagy or mitophagy (Zhu et al., 2023). This is particularly important because it allows apparently unrelated toxic effects to be discussed under a common mechanistic umbrell (Peng et al., 2022). For instance, a drug that perturbs mitochondrial quality control in neural tissue, a therapy that triggers oxidative injury in hepatocytes, and an exposure that destabilizes lipid metabolism in cardiac tissue may all converge on a common pattern of insufficient stress adaptation (Zhou et al., 2023). Read together, these findings reveal mechanistic continuity behind otherwise heterogeneous clinical endpoints (Huang et al., 2022; Akhtar et al., 2023; Zhuo et al., 2023).

Supportive evidence further indicates that host factors strongly modify metabolic toxicity expression (Lu et al., 2021; Chen Y. et al., 2022; Rahman et al., 2022). Pre-existing metabolic disease, obesity, chronic inflammation, aging, organ dysfunction, and polypharmacy can all reduce reserve capacity and magnify the impact of otherwise modest mitochondrial insults (Dai et al., 2020; Zhang et al., 2020; Huang et al., 2021). Moreover, disease-state metabolism itself may interact with pharmacotherapy in ways that either unmask toxicity or shift it toward particular tissues (Duan et al., 2020; Gao et al., 2020; Yi et al., 2020). In practice, this means that risk is not determined solely by drug class but by the alignment of drug mechanism with the patient’s baseline metabolic fragility (Zhao et al., 2019).

Across studies, a small number of recurring metabolic patterns stand out (Dong et al., 2023; Jiang et al., 2024c; Zhu et al., 2025). Mitochondrial dysfunction, oxidative stress, altered substrate metabolism, defective mitophagy, and ferroptosis-associated injury recur across otherwise unrelated drug classes (Peng et al., 2022; Li Q. et al., 2023; Zhu et al., 2023). These mechanisms then reappear clinically through liver, heart, brain, kidney, and skeletal muscle involvement, often with corresponding implications for biomarker monitoring, dose adjustment, rescue strategies, or susceptibility assessment (Zhao et al., 2019; Huang et al., 2022; Gao et al., 2023).

Representative metabolic pathways, associated toxic phenotypes, and mechanistic interpretations are summarized in Table 2, which integrates evidence across diverse therapeutic contexts while avoiding redundancy with the main text.

**TABLE 2:** Representative metabolic and mitochondrial toxicity mechanisms across therapeutic systems.

Mechanistic Motif	Representative Model	Clinically Recognized Toxicity	Mechanistic Basis	Biomarker Or Prediction Angle	Monitoring Or Management Implication	Candidate References
Reactive metabolite formation and mitochondrial oxidative stress	Acetaminophen overdose	Acute liver injury and acute liver failure	CYP-mediated NAPQI formation depletes glutathione; mitochondrial protein adducts, oxidative stress, JNK activation, and necrotic cell death follow	Exposure history, acetaminophen concentration, ALT/AST, INR, bilirubin, lactate; DILI stopping-rule logic	Early N-acetylcysteine treatment and liver-failure risk triage	Acetaminophen hepatotoxicity mechanistic studies; FDA DILI guidance
Impaired fatty-acid oxidation and urea-cycle stress	Valproate	Hepatotoxicity, hyperammonemia, encephalopathy; higher risk in POLG-related mitochondrial disease	Interference with mitochondrial beta-oxidation and ammonia metabolism; genetically susceptible mitochondrial disease increases risk	POLG/mitochondrial disease history, ammonia, liver enzymes, mental-status change	Avoidance or extreme caution in known mitochondrial disease; symptom-triggered ammonia and liver-function monitoring	Valproate mitochondrial toxicity literature; POLG/VPA safety literature
Mitochondrial ribosomal inhibition	Linezolid	Lactic acidosis, myelosuppression, peripheral or optic neuropathy	Inhibition of mitochondrial protein synthesis due to similarity between bacterial and mitochondrial ribosomes	Treatment duration, lactate when symptomatic, CBC trend, neurologic/visual symptoms	Limit prolonged exposure where possible; monitor CBC and neurologic/visual toxicity during extended therapy	linezolid mitochondrial toxicity studies (Garrabou et al., 2007)
Cardiomyocyte mitochondrial injury and DNA-damage stress	Doxorubicin and other anthracyclines	Cancer therapy-related cardiac dysfunction and heart failure	Oxidative injury, mitochondrial dysfunction, and topoisomerase II beta-mediated cardiomyocyte DNA damage	Baseline LVEF/GLS, troponin, natriuretic peptides, cumulative anthracycline dose	Risk-adapted echocardiographic/biomarker surveillance; dexrazoxane or cardioprotective strategies in selected high-risk contexts	2022 ESC cardio-oncology guideline (Lyon, 2022)
Mitochondrial DNA depletion	Older nucleoside reverse transcriptase inhibitors	Lactic acidosis, hepatic steatosis, myopathy, neuropathy	Impaired mitochondrial DNA polymerase gamma reduces oxidative phosphorylation capacity	Lactate, liver enzymes, neuromuscular symptoms, exposure to older high-risk NRTIs	Prefer safer regimens where possible; symptom-triggered metabolic and hepatic assessment	HIV mitochondrial toxicity literature
Skeletal-muscle exposure and energy-stress susceptibility	Statins	Myopathy, myositis, rhabdomyolysis	Dose/exposure-dependent muscle injury; risk increased by drug interactions, renal impairment, older age, and selected transporter variants	CK when symptomatic, renal function, drug-drug interactions, high-dose or high-exposure statin use	Dose adjustment, interaction avoidance, alternative statin selection, renal-risk management	Statin safety statements; DIRA
Tissue accumulation, phospholipidosis, and endocrine-metabolic disruption	Amiodarone	Hepatotoxicity, pulmonary toxicity, thyroid dysfunction	Long tissue half-life, mitochondrial injury, phospholipidosis, and iodine-mediated thyroid effects	Liver enzymes, thyroid function, pulmonary symptoms/imaging, cumulative exposure	Scheduled thyroid/liver monitoring and prompt pulmonary evaluation when symptomatic	Amiodarone safety reviews/guidance
Renal clearance-dependent lactate handling	Metformin during renal impairment or acute illness	Metformin-associated lactic acidosis, rare but severe	Reduced clearance plus acute tissue hypoxia or renal dysfunction promotes lactate accumulation	eGFR, acute illness, hypoxia/sepsis, lactate and acid-base status when symptomatic	eGFR-based prescribing and temporary interruption during severe illness or high-risk contrast/surgical scenarios	FDA metformin renal guidance; clinical reviews
Drug-induced metabolic pathway remodeling	Nilotinib and selected TKIs	Hyperglycemia, dyslipidemia, vascular events	Kinase-pathway interference with metabolic signaling and endothelial function	Glucose, lipid profile, cardiovascular risk factors, treatment duration	Metabolic monitoring and cardiovascular risk management during long-term targeted therapy	Cardio-oncology guidance; TKI safety literature
Preclinical mitochondrial liability and translational biomarker integration	Candidate drugs with mitochondrial respiration or membrane-potential effects	Potential hepatotoxicity, cardiotoxicity, or myotoxicity during development	Mitochondrial stress can amplify organ-specific susceptibility under exposure, inflammation, transporter inhibition, or genetic risk	Mitochondrial respiration assays, DILI biomarkers, transporter data, exposure margins, omics signatures	Integrate mechanistic assays with exposure and clinical biomarker plans during safety assessment	FDA DILI guidance; toxicology biomarker literature

The forward-looking conclusion is that metabolic toxicity should increasingly be anticipated rather than discovered after injury becomes clinically obvious. As more therapies intersect with immunometabolism, mitochondrial signaling, and redox biology, safety assessment will need to move beyond standard organ chemistry panels toward earlier detection of stress phenotypes and adaptive failure (Meng et al., 2020; Chen X. et al., 2021; Zhao et al., 2025; Li et al., 2026). Taken together, metabolic and mitochondrial toxicity provides a unifying mechanistic layer across multiple adverse-event phenotypes and should be regarded as a major organizing concept in modern pharmacotherapy safety rather than a niche toxicology subsection.

## 4 Immune-mediated toxicity

Immune toxicity is clinically distinctive because therapeutic benefit and inflammatory injury may arise from the same intervention. The problem is not merely excessive inflammation, but loss of control over where, when, and how immune activation is expressed.

Few areas of current drug safety are as clinically consequential as immune-mediated toxicity (Qi et al., 2022; Jayathilaka et al., 2024; Mizuno et al., 2025; Xu L. et al., 2025; Yamauchi and Yabe, 2025). What first became apparent with immune checkpoint blockade now extends across biologics, cell therapies, antibody-based interventions, and combination regimens that reshape immune-cell activation, trafficking, or cytokine balance. The central difficulty is that the mechanisms responsible for durable disease control can also generate pneumonitis, colitis, hepatitis, myocarditis, endocrinopathy, neurologic syndromes, dermatologic reactions, and multisystem inflammatory injury when tolerance breaks down.

Immune-mediated toxicity is not simply “more inflammation,” but a misdirected or excessive immune reorganization that escapes normal spatial and temporal regulation (Zhou et al., 2019; Gan et al., 2022; Gazzano et al., 2022; Tsukamoto et al., 2022; Fang et al., 2025). In clinical terms, this can appear as pneumonitis, colitis, hepatitis, myocarditis, endocrinopathy, neurologic syndromes, dermatologic reactions, or multisystem inflammatory injury. In mechanistic terms, however, these diverse manifestations are linked by shared failures of tolerance, altered effector-cell behavior, cytokine disequilibrium, or aberrant antigen recognition. This dual clinical-mechanistic framing is important because it preserves coherence even when the organ phenotypes are heterogeneous.

Checkpoint inhibitor experience remains the clearest reference point for immune-mediated toxicit (Hong and Ding, 2019; Wang et al., 2019; Xiao et al., 2020). These studies show that immune-related adverse events are not random idiosyncrasies but patterned toxic syndromes shaped by treatment class, host immune context, tissue susceptibility, and prior autoimmune background. Reviews and pooled analyses further indicate that incidence alone is an incomplete metric; severity distribution, timing, organ clustering, response to immunosuppression, and recurrence after rechallenge often matter just as much in the clinical setting. What matters most is not enumeration alone but the link between pathogenesis, recurrence risk, and management implications.

A second core theme is that immune-mediated toxicity often reflects a balance problem rather than a binary on/off event (Fu et al., 2021; Pi et al., 2024; Tan S. et al., 2024; Gao et al., 2025; Tang L. et al., 2025). Therapies that activate cytotoxic lymphocytes, alter macrophage behavior, or redistribute inflammatory set points may improve disease control while simultaneously lowering the threshold for off-tumor or off-target tissue injury. The literature on myocarditis, thyroid dysfunction, pulmonary toxicity, gastrointestinal inflammation, and delayed hematologic syndromes shows that organ injury may arise when immune amplification outpaces regulatory containment. This connection between pathophysiology and management matters because early recognition, graded immunosuppression, retreatment decisions, and biomarker monitoring all depend on understanding toxicity as dysregulated immune balance rather than as isolated organ inflammation.

Supportive studies further indicate that risk is shaped by baseline autoimmune history, combination therapy, and prior inflammatory burden (Zhang N. et al., 2022; Sepasi et al., 2023; Shen et al., 2023). Some patients appear predisposed because of pre-existing immune fragility, whereas others develop severe toxicity only after sequential or combined interventions magnify immune activation beyond a compensable threshold. The same treatment can therefore produce mild endocrinopathy in one patient and life-threatening myocarditis or pneumonitis in another. Susceptibility is best understood as layered and context-dependent rather than as a fixed patient trait.

Several patterns recur across the immune-mediated safety literature (Wang et al., 2018; Zhou et al., 2020; Morikawa et al., 2025; Yan et al., 2025; Ma et al., 2026). Mechanism, dominant organ pattern, time to onset, standard management approach, and rechallenge feasibility often move together rather than independently. Similar clustering appears when therapy class is considered alongside syndrome type and severity modifiers. This evidence base is most convincing when it shows how shared immunobiology produces recognizable clinical syndromes with consistent management consequences.

Taken together, immune-mediated toxicity is now a mature and indispensable safety domain rather than a special topic confined to immuno-oncology. The field is moving toward prediction through biomarkers and immune phenotyping, but it is not yet at the stage where severe events can be consistently prevented. Immune-mediated toxicity is best understood as a translational interface between immunobiology and safety management: understanding the mechanism is not optional, because mechanism directly shapes surveillance, steroid use, treatment interruption, and long-term patient counseling.

## 5 Organ-specific manifestations

Clinicians usually encounter drug toxicity first as an organ problem rather than as a mechanistic abstraction. Hepatic, cardiac, renal, neurologic, pulmonary, and hematologic patterns therefore remain indispensable for translating safety science into bedside recognition.

Organ-specific manifestations provide the clinically recognizable face of drug toxicity, even though the underlying mechanisms are often shared across therapeutic classes and biologic systems (Chen et al., 2020b; Annawald et al., 2024; Karschnia et al., 2025; Nielsen et al., 2025). In practice, safety signals enter clinical awareness through organ-patterned syndromes such as hepatotoxicity, cardiotoxicity, nephrotoxicity, neurotoxicity, pulmonary injury, hematologic complications, or gastrointestinal damage (Fan et al., 2020). A productive review strategy is therefore to treat organ manifestations not as isolated endpoints, but as the convergence point where exposure, mechanism, host susceptibility, and surveillance quality become visible to clinicians.

Organ-based classification remains clinically necessary even when mechanistic classification is increasingly sophisticated (Zhang M. et al., 2024). Clinicians make treatment decisions through organ signals: rising transaminases, arrhythmia, creatinine change, neuropathy, dyspnea, bleeding, or altered mental status (Tao et al., 2024). As a result, an organ-specific discussion remains essential for translational relevance, because it links abstract toxicology to actionable bedside patterns. At the same time, organ toxicity should not be interpreted as mechanistically isolated, since many organ manifestations are downstream expressions of broader processes such as oxidative stress, endothelial dysfunction, immune activation, mitochondrial failure, or coagulation perturbation (Wei Y. et al., 2023).

Across comparative studies and clinical reports, hepatic, cardiac, renal, and neurologic toxicities remain the dominant anchor points for organ-specific safety synthesis (Zhao et al., 2022; Lu R. et al., 2023; Zhang et al., 2023). Hepatic injury is often discussed through idiosyncratic versus predictable mechanisms, metabolism-dependent injury, cholestatic or hepatocellular patterns, and interaction with gut-liver signaling or inflammatory status. Cardiac toxicity is frequently framed around electrophysiologic instability, myocardial injury, and cumulative cardiometabolic stress, particularly under targeted therapy and multimorbidity conditions (Tian et al., 2018). Renal toxicity often reflects altered perfusion, tubular exposure, and transporter-mediated effects (Lu R. et al., 2023; Zhang et al., 2023; Tao et al., 2024; Zhang M. et al., 2024), whereas neurologic toxicity may present through seizure risk, cognitive change, neuropathy, or treatment-related neuroinflammation (Wei Y. et al., 2023; Karschnia et al., 2025; Nielsen et al., 2025). Organ-based framing remains useful not because it simplifies the science, but because it creates a clinically legible map of where toxicity ultimately manifests.

Another important point is that organ manifestation patterns can reveal hidden mechanistic differences between therapies that would otherwise appear similar (Wang Y. et al., 2020). Two drugs may both be labeled “safe” in trial summaries, yet differ substantially in whether they concentrate risk in liver chemistry abnormalities, cardiovascular instability, or delayed neurologic intolerance. Comparative studies and observational cohorts are especially useful here because they expose the practical asymmetry of adverse-event burdens across regimens and populations. This allows the discussion to move from a generic list of toxicities toward a comparative narrative: which organs are dominant risk targets for which therapeutic contexts, and how consistently these patterns recur across evidence layers.

Supportive literature further indicates that organ manifestations are shaped by baseline vulnerability and therapeutic setting (Zhang et al., 2019; Bai et al., 2020; Zhao et al., 2020). Older adults, patients with chronic kidney disease, liver disease, cardiovascular disease, or cancer-related frailty often experience the same pharmacologic insult differently because reserve capacity and exposure handling differ profoundly (Feng et al., 2021; Qin et al., 2021; Zhou et al., 2021). Clinical context, including oncologic, intensive care, or chronic outpatient settings, also influences how rapidly toxicity is detected and managed (Li R. et al., 2020). This means that organ-specific toxicity should not be treated as a purely pharmacologic phenomenon; it is also a context-dependent clinical phenomenon, and that distinction is essential for interpreting trial data alongside real-world safety observations (Huang et al., 2020).

Organ-based safety studies repeatedly converge on a limited number of clinically useful comparisons. Across drug classes, the same questions recur: which organ systems are most vulnerable, what adverse events dominate early presentation, which mechanisms are most plausible, and when monitoring should escalate. These comparisons become especially informative when organ liability is linked back to class-specific patterns and practical thresholds for intervention. Representative organ-specific toxicity patterns, underlying mechanisms, and associated clinical contexts are summarized in Table 3.

**TABLE 3:** Representative organ-specific toxicity patterns and underlying mechanisms across therapeutic systems.

Organ System	Clinically Established Model	Representative Toxicity	Dominant Mechanism	Predictive Or Monitoring Implication	Candidate References
Liver	Acetaminophen	Dose-dependent acute liver injury/failure	NAPQI-mediated oxidative and mitochondrial injury	Acetaminophen level, ALT/AST/INR, lactate, early antidote treatment	Acetaminophen hepatotoxicity literature; FDA DILI guidance
Liver	Isoniazid and antituberculosis therapy	Idiosyncratic or metabolic drug-induced liver injury	Reactive metabolites, immune susceptibility, host metabolic risk	Baseline liver risk review, symptom education, liver enzyme monitoring in high-risk patients	LiverTox; TB treatment guidance
Heart	Anthracyclines	Left ventricular dysfunction, heart failure	Mitochondrial injury and topoisomerase II beta-mediated cardiomyocyte damage	Echocardiography, troponin/natriuretic peptide surveillance, cumulative-dose risk	2022 ESC cardio-oncology guideline (Lyon, 2022)
Heart	QT-prolonging drugs (sotalol, methadone, antipsychotics, macrolides)	Torsades de pointes and sudden cardiac death risk	hERG potassium-channel blockade and repolarization delay	Baseline QTc/electrolytes, interaction review, ECG monitoring when indicated	QT/TdP guidance; DIQTA (Li et al., 2022b)
Kidney	Cisplatin	Acute kidney injury, tubular dysfunction, electrolyte wasting	Proximal tubular uptake, oxidative stress, inflammation, and apoptosis	Baseline renal function, hydration protocols, magnesium/electrolyte monitoring, dose adjustment	Cisplatin nephrotoxicity reviews/guidance
Kidney/ear	Aminoglycosides (gentamicin)	Nephrotoxicity and ototoxicity	Proximal tubular and inner-ear accumulation with mitochondrial/oxidative injury	Therapeutic drug monitoring, renal-function monitoring, duration limitation	Aminoglycoside safety literature (Francke et al., 2021)
Lung	Amiodarone	Interstitial pneumonitis/fibrosis	Tissue accumulation, phospholipidosis, oxidative injury	Baseline respiratory history, imaging/PFTs when symptomatic, early discontinuation when suspected	Amiodarone pulmonary toxicity literature
Skin	Carbamazepine	Stevens-Johnson syndrome/toxic epidermal necrolysis	HLA-associated immune recognition, especially HLA-B*15:02 in high-risk ancestry groups	Pre-treatment HLA screening in indicated populations	CPIC HLA/carbamazepine guideline
Immune/endocrine organs	Immune checkpoint inhibitors	Thyroiditis, hypophysitis, colitis, pneumonitis, myocarditis	Immune disinhibition and tissue-specific autoimmunity	Baseline and symptom-triggered endocrine/cardiopulmonary monitoring; irAE algorithms	Salem 2018; Nguyen 2022; oncology irAE guidelines (Salem et al., 2018; Nguyen, 2022)
Blood/bone marrow	Thiopurines	Severe myelosuppression	TPMT/NUDT15 loss-of-function variants increase active thioguanine nucleotide exposure	Pre-treatment TPMT/NUDT15 genotyping or phenotyping and genotype-guided dosing	CPIC TPMT/NUDT15 guideline
Muscle	Statins	Myopathy and rhabdomyolysis	Exposure-related muscle injury, drug interactions, renal dysfunction, genetic susceptibility	Symptom-triggered CK, interaction review, dose/risk adjustment	Statin safety statements; DIRA
Nervous system	Linezolid	Peripheral/optic neuropathy, encephalopathy with lactic acidosis	Mitochondrial protein synthesis inhibition	Duration-limited use where possible; neurologic/visual and lactate monitoring in prolonged therapy	linezolid mitochondrial toxicity studies (Garrabou et al., 2007)

Taken together, organ-specific manifestations will remain indispensable in safety writing, but they are most informative when interpreted through cross-organ mechanistic integration rather than siloed toxicology categories (Liu N. et al., 2025). That shift matters because future safety practice will likely rely on linking early biomarker change, organ reserve, and therapeutic mechanism before irreversible injury develops (Badran et al., 2025; Chu et al., 2025). Organ-based patterns still provide the clearest entry point for clinicians, but their explanatory value increases when they are connected back to broader pharmacologic mechanisms and risk-management strategies. Clinicians should be aware that similar organ-specific toxicity patterns may arise from distinct underlying mechanisms, and that accurate interpretation requires integration of clinical presentation with mechanistic context.

## 6 Clinical evidence and real-world safety

No matter how persuasive the mechanism, a safety claim remains incomplete until it survives comparison across trials, pooled analyses, and routine clinical use. Much of the uncertainty in contemporary pharmacotherapy lies precisely in that gap.

Early trial datasets rarely capture the full safety profile of biologics, antibody-drug conjugates, cell therapies, and highly selective targeted agents (Bate and Luo, 2022; Brown et al., 2025; Liu S. et al., 2025; Youssef et al., 2025; Rathod et al., 2026). Rare events, delayed toxicities, subgroup-specific harms, and utilization-pattern effects often become legible only after therapies move into broader clinical settings. For that reason, modern safety assessment depends on a layered evidence architecture in which randomized trials establish initial tolerability, systematic reviews and network meta-analyses compare competing regimens, and pharmacovigilance or cohort datasets detect signals that were not fully visible during registration studies (Bettuzzi et al., 2021; Wen et al., 2022; Jiang et al., 2025).

What emerges most clearly from this literature is the value of evidence synthesis for understanding how safety profiles differ across therapeutic classes, diseases, and treatment settings (Chen et al., 2023; Jiang K. et al., 2024; Niu et al., 2025). Meta-analyses in autoimmune disease, oncology, cardiometabolic medicine, perioperative analgesia, and infectious disease repeatedly show that pooled estimates can clarify whether adverse events represent class effects, dose effects, exposure-duration effects, or context-dependent toxicities emerging only in high-risk populations (Feng H. et al., 2023; Yang et al., 2023; Wang Y. et al., 2024; Cai B. et al., 2025). Network meta-analytic approaches are especially useful when head-to-head trials are sparse, because they allow simultaneous comparison of multiple regimens while preserving clinically interpretable endpoints such as bleeding, infection, cardiovascular complications, or treatment discontinuation (Fu et al., 2023; Lu D.-H. et al., 2023; Dang et al., 2024).

Randomized and multicenter comparative studies remain indispensable because they anchor the hierarchy of evidence and define the denominator against which later real-world concern should be interpreted (Jiang et al., 2022; Wang M. et al., 2022; Wang X. et al., 2022; Hou et al., 2023; Li Y. et al., 2023). In this regard, trials and pooled analyses on anticoagulants, sedatives, anti-inflammatory biologics, antibiotics, and supportive care interventions illustrate that safety is not a single endpoint but a composite domain spanning hemodynamic instability, bleeding, infectious events, organ toxicity, procedural complications, and treatment-emergent intolerance (Cheng et al., 2020; Lang et al., 2020; Su et al., 2020; Wang W. et al., 2020; Xu et al., 2022). The major contribution of these studies is not merely the reporting of event rates, but the standardization of how risk is framed across dose, route, concomitant medication, and patient subgroup, thereby creating a scaffold for later observational validation (Zeng et al., 2020; Chen Y. et al., 2021; Yang et al., 2021).

Real-world evidence then extends this scaffold into the messier but clinically decisive environment of routine care (Zheng et al., 2020; Li Z. et al., 2025; Shi et al., 2025). Retrospective cohorts, pharmacovigilance analyses, and multicenter observational studies are particularly useful for detecting low-frequency toxicities, delayed adverse reactions, and utilization-pattern effects that depend on age, comorbidity, polypharmacy, organ dysfunction, or off-label combination use (Tian et al., 2022b; Liu et al., 2024d; Guo J. et al., 2025; Huang et al., 2025). Such studies are also crucial for drugs whose post-marketing use rapidly expands beyond the populations originally enrolled in trials, because they reveal how safety changes when monitoring intensity falls, indication boundaries widen, and prescribing behavior becomes heterogeneous (Li L. et al., 2023; Ren J.-W. et al., 2024; Tan J. et al., 2024). For this reason, the strongest real-world safety literature should not be viewed as inferior to trial data, but as a complementary layer that helps convert regulatory tolerability into clinically actionable risk knowledge (Lin et al., 2025; Luo et al., 2025; Zhang J. et al., 2025).

Several recurring contrasts shape this evidence base (Su et al., 2023; Zhang and Chen, 2024). Randomized trials and meta-analyses define the strength of comparative inference, whereas retrospective cohorts, pharmacovigilance datasets, and umbrella reviews extend detection into lower-frequency and delayed events (Ma Q. et al., 2022). Safety endpoints also cluster in recognizable ways, including cardiotoxicity, infection, bleeding, hepatotoxicity, medication error, and discontinuation due to adverse events. These patterns look different across perioperative care, chronic inflammatory disease, cancer therapy, pediatric pharmacotherapy, and vulnerable populations such as older adults, pregnant patients, and patients with organ impairment (Dong et al., 2022). In real-world context, safety interpretation depends as much on evidence setting and population context as on the drug itself. Representative examples of trial-based, observational, and pharmacovigilance-derived safety evidence are summarized in Table 4.

**TABLE 4:** Representative examples of trial-based, observational, and pharmacovigilance-derived safety evidence in contemporary pharmacotherapy.

Evidence Layer	Typical Data Source Or Design	Safety Question Best Addressed	Strength	Main Limitation	Representative Example	Candidate References
Mechanistic toxicology	*In vitro* assays, animal models, omics, mitochondrial and transporter assays	Is there a biologically plausible toxicity pathway?	Detects mechanism before broad human exposure	Translation to human risk can be uncertain	Mitochondrial liability assays for DILI or myotoxicity	FDA DILI guidance; mechanistic toxicology reviews
Early-phase clinical trials	Phase I/II dose-escalation and dose-expansion studies	What dose/exposure produces early tolerability signals?	Controlled exposure and detailed monitoring	Limited size and selected participants	Early hematologic toxicity with PARP inhibitors or targeted oncology agents	Drug labels; oncology trial reports
Randomized comparative safety trials	RCTs designed or powered for safety or benefit-risk comparison	Does one therapy carry higher risk than an active comparator in a defined population?	Strong comparator control and prespecified endpoints	Expensive, slow, still limited generalizability	ORAL Surveillance: tofacitinib versus TNF inhibitors in RA risk population	ORAL Surveillance; FDA JAK warning
Pharmacogenomic validation trials	Prospective genotype-guided treatment studies	Can pre-treatment testing prevent a serious adverse reaction?	Directly links biomarker to risk mitigation	Applies only to well-validated marker-drug pairs	PREDICT-1: HLA-B*57:01 screening to prevent abacavir hypersensitivity	Mallal et al. (2008)
Meta-analysis and systematic review	Aggregated trial or observational literature	What is the overall risk estimate across studies?	Improves precision and summarizes heterogeneity	Dependent on source-study quality and reporting	EGFR inhibitor skin toxicity or GLP-1RA biliary risk synthesis	Topic-specific meta-analyses
Spontaneous reporting systems	FAERS, VigiBase, EudraVigilance	Is there a disproportionality signal for rare or unexpected events?	Broad postmarketing coverage and early signal generation	Reporting bias, missing denominators, causality limits	ICI myocarditis/cardiovascular irAEs detected through pharmacovigilance	Salem et al. (2018); Nguyen (2022)
Active surveillance and claims/EHR networks	Sentinel, national registries, insurance claims, EHR cohorts	Does a signal persist in population-level routine care?	Uses large denominators and comparative designs	Confounding and coding limitations require careful design	FDA Sentinel active medical product safety surveillance	FDA Sentinel Initiative (U.S. Food and Drug Administration, 2024)
Real-world clinical cohorts and registries	Disease registries, hospital cohorts, specialty databases	How does toxicity present in broader patients with comorbidities?	Captures practice heterogeneity and vulnerable groups	Residual confounding and variable ascertainment	ICI myocarditis cohorts; antituberculosis DILI cohorts	Clinical cohort studies
Artificial intelligence and computational pharmacovigilance	NLP, machine learning, knowledge graphs, signal-prioritization tools	Can heterogeneous data be transformed into earlier risk detection or stratification?	Scales detection across unstructured and multimodal data	Requires validation, transparency, and bias control	NLP-based irAE detection; DILI prediction models	AI pharmacovigilance literature
Regulatory risk-management frameworks	Labeling, boxed warnings, REMS/RMP, ICH pharmacovigilance planning	How should known or potential risks be monitored, communicated, and mitigated?	Connects evidence to implementation	Region-specific requirements and evolving standards	ICH E2E pharmacovigilance planning; FDA safety communications	ICH E2E; FDA guidance (International Council for Harmonisation, 2004)

Another important point is that safety evidence should be interpreted as a dynamic continuum rather than a static label attached to a drug. Signal refinement now increasingly depends on artificial intelligence, machine learning, and large spontaneous-reporting databases, which can accelerate case finding but also amplify reporting bias, publicity effects, and confounding by indication if not paired with disciplined clinical interpretation. The most persuasive studies in this domain are therefore those that integrate pharmacovigilance signals with external evidence from trials, biologic plausibility, and disease-specific context, rather than treating disproportionality alone as proof of causality.

Taken together, the literature discussed here supports a modern safety framework in which randomized evidence defines baseline tolerability, evidence synthesis compares regimens across therapeutic classes, real-world datasets identify uncommon or delayed risks, and advanced pharmacovigilance tools refine post-marketing surveillance. The forward-looking challenge is to turn this layered evidence base into predictive safety models that guide drug selection, monitoring intensity, rechallenge decisions, and individualized counseling before serious toxicity occurs. That shift from retrospective event counting to anticipatory risk management is likely to define the next stage of modern pharmacotherapy safety research.

## 7 Risk prediction and management

Risk management is where safety evidence either becomes clinically actionable or remains descriptive. The practical difficulty is not simply identifying toxicities, but deciding which signals matter early enough to change outcomes. Clinicians should therefore prioritize early risk stratification and continuous monitoring to identify patients at increased risk and intervene before irreversible injury occurs. As illustrated in Figure 1, risk prediction and monitoring serve as the central link between mechanistic understanding and clinical decision-making.

Once serious toxicity is recognized as a predictable rather than purely reactive problem, the logic of safety research changes (Fu et al., 2020; Ni et al., 2021). The central question becomes whether harm can be anticipated, stratified, and mitigated before clinical deterioration occurs. That challenge is intensified by targeted agents, biologics, cell therapies, combination regimens, and long-term maintenance strategies, all of which require proactive frameworks that link baseline susceptibility, exposure characteristics, biomarker evolution, and post-marketing signal detection.

Prediction depends on combining multiple evidence layers rather than relying on any single marker or study type (Hu et al., 2018; Fan et al., 2019; Xin et al., 2019; Tian et al., 2023a). Clinical risk factors, prior organ dysfunction, age, frailty, polypharmacy, inflammatory status, and comorbidity remain foundational. However, these variables become meaningfully actionable only when they are interpreted together with treatment-specific evidence from trials, pharmacovigilance databases, real-world cohorts, and mechanistic studies that clarify why certain patients are more vulnerable to particular toxic effects. This layered approach is especially important because high-risk patients are often underrepresented in registration trials but overrepresented in real practice.

The core literature suggests three major components of effective risk prediction: pre-treatment stratification, dynamic monitoring, and signal integration (Wen et al., 2019; Li S. et al., 2022; Xu H. et al., 2025). Representative studies, tools, and interventions across different evidence settings, along with their associated risk and safety focus and relevance to prediction or management, are summarized in Table 5. The first includes disease burden, organ reserve, baseline laboratory features, prior toxicity history, and concomitant medication exposure. The second depends on serial biomarkers, symptom tracking, imaging, or structured surveillance to identify deviation from expected recovery or tolerability patterns. The third requires pharmacovigilance alerts, spontaneous-reporting clusters, and observational safety trends to be interpreted in light of clinical plausibility rather than treated as isolated warnings. These components are best understood not as separate administrative boxes, but as a continuous risk-management cycle spanning pre-treatment, on-treatment, and post-signal response.

**TABLE 5:** Representative risk prediction models, pharmacovigilance signals, and management frameworks in pharmacotherapy safety.

Risk Model Or Management Approach	Drug Or Toxicity Example	Prediction Or Management Target	Actionable Marker Tool Or Intervention	Clinical Implication	Candidate References
Pre-treatment HLA screening	Abacavir hypersensitivity	Prevent immune-mediated hypersensitivity	HLA-B*57:01 testing; avoid abacavir in carriers	Clear example of a validated pharmacogenomic risk-mitigation strategy	Mallal 2008; NCBI Medical Genetics Summary (Mallal et al. (2008)
Pre-treatment HLA screening	Carbamazepine-induced SJS/TEN	Prevent severe cutaneous adverse reaction	HLA-B*15:02 testing in high-risk ancestry groups; consider HLA-A*31:01 depending on context	Links ancestry-aware pharmacogenomics with prescribing safety	CPIC HLA/carbamazepine guideline
Pharmacogenomic dose adjustment	Thiopurine myelosuppression	Prevent severe leukopenia/myelosuppression	TPMT and NUDT15 genotyping or phenotyping; genotype-guided starting dose	Mature model for translating genotype into dose selection	CPIC TPMT/NUDT15 guideline
Exposure and organ-function adjustment	DOACs, aminoglycosides, metformin	Bleeding, nephrotoxicity, or lactic acidosis in renal impairment	eGFR, age, interacting drugs, therapeutic drug monitoring where applicable	Shows that prediction often combines PK exposure and clinical covariates	FDA renal dosing guidance; class-specific labels
Cardio-oncology surveillance	Anthracycline cardiotoxicity	Early detection of cancer therapy-related cardiac dysfunction	Baseline echocardiography, GLS, troponin, natriuretic peptides, cumulative dose	Converts organ toxicity into scheduled surveillance and intervention	2022 ESC cardio-oncology guideline (Lyon, 2022)
QT risk stratification	QT-prolonging drugs	Torsades de pointes prevention	Baseline QTc, electrolytes, renal/hepatic function, interacting QT drugs	Useful for bedside and digital decision-support workflows	QT guidance; DIQTA (Li et al., 2022b)
Immune-related adverse event algorithms	ICI colitis, myocarditis, endocrinopathy, pneumonitis	Early recognition and graded management	Symptom triage, organ-specific labs/imaging, CTCAE grading, corticosteroid/immunosuppressive algorithms	Shifts toxicity management from reactive recognition to protocolized care	ASCO/ESMO/NCCN irAE guidance; pharmacovigilance studies
DILI signal detection and stopping rules	DILI across hepatotoxic drugs	Identify serious hepatocellular injury risk	ALT/AST, bilirubin, Hy’s law logic, symptoms, rechallenge avoidance	Connects biochemical signals with regulatory and clinical risk control	FDA DILI guidance
Postmarketing active surveillance	Newly approved drugs or expanded indications	Detect rare, delayed, or population-specific adverse events	Sentinel-style distributed data networks, claims/EHR analyses	Complements spontaneous reporting with denominator-based assessment	FDA Sentinel Initiative (U.S. Food and Drug Administration, 2024)
AI/NLP pharmacovigilance	irAEs, DILI, QT prolongation, polypharmacy-related events	Earlier signal detection or case identification	NLP extraction from clinical notes, disproportionality prioritization, model validation	Promising but should be presented as decision support, not replacement for clinical causality assessment	AI pharmacovigilance literature
Patient-level deprescribing and medication review	Older adults with polypharmacy	Falls, bleeding, anticholinergic burden, inappropriate medication use	Beers Criteria, STOPP/START, renal function, frailty, drug-drug interaction review	Makes risk management practical in routine pharmacotherapy	Geriatric prescribing criteria/guidelines
Risk communication and regulatory action	JAK inhibitors, fluoroquinolones, opioids	Translate emerging evidence into label warnings and prescribing restrictions	Boxed warnings, medication guides, REMS/RMP when appropriate	Shows that risk assessment ends in communication and clinical behavior change	FDA safety communications; ICH E2E (U.S. Food and Drug Administration, 2021)

Biomarker-informed management is becoming particularly important because many serious adverse reactions are preceded by measurable but underused early changes (Wang M.-G. et al., 2022; Zhang H. et al., 2022). Depending on therapeutic context, these may include inflammatory proteins, troponin, liver enzymes, kidney function trends, coagulation shifts, immune-cell patterns, or treatment-specific molecular indicators. The literature repeatedly shows that isolated biomarker abnormalities are often nonspecific, but serial change combined with mechanistic context substantially improves risk discrimination. Single biomarkers should therefore not be overstated and are better framed as components of structured surveillance algorithms.

Another major theme in this domain is that management must be proportional rather than uniform (Gao et al., 2019; Song et al., 2022; Cao et al., 2026; Shojaei et al., 2026). Not all detected toxicity signals require discontinuation, and not all apparently mild events are benign. Effective management depends on distinguishing reversible pharmacodynamic effects from evolving organ injury, class effects from patient-specific events, and manageable inflammatory syndromes from toxicities likely to recur with rechallenge. This is where comparative evidence, consensus guidance, and real-world outcome studies become particularly valuable, because they help define which interventions are associated with safer continuation, dose modification, prophylaxis, retreatment, or transition to alternative regimens.

The support literature also highlights the growing role of pharmacovigilance informatics and machine learning in early signal prioritization (Wu et al., 2024; Chen S. et al., 2025; He et al., 2025; Kantagba et al., 2025; Tang X. et al., 2025; Tao et al., 2025; Xie et al., 2025). Large safety databases can detect emerging patterns faster than conventional clinical observation, especially for rare, delayed, or combination-related adverse reactions. Yet the key limitation is that such systems can overcall noise, publicity bias, channeling bias, or duplicate signal inflation if not paired with disciplined adjudication. This tension is useful because it presents AI-enabled pharmacovigilance as an important accelerant for risk management, but not a substitute for causal reasoning, expert review, and clinically grounded validation.

A final pattern emerges when baseline risk factors, early signals, monitoring intensity, and management responses are considered together (Tian et al., 2022a; Singla et al., 2024; Zeng J. et al., 2024; Su et al., 2025). Across therapeutic classes, the same practical sequence recurs: susceptibility is identified, early warning signs are recognized, surveillance is intensified, and intervention is calibrated before irreversible injury develops. Related studies also show that escalation thresholds, de-escalation strategies, and rechallenge decisions tend to cluster by toxicity pattern rather than by single drugs alone. The value of this evidence lies in turning heterogeneous safety observations into decision pathways that can actually guide practice.

Taken together, future progress in pharmacotherapy safety will depend less on simply accumulating more adverse-event data and more on converting heterogeneous evidence into actionable decision pathways (Li D. et al., 2022; Yang et al., 2022). Risk prediction should move toward integrated models that combine baseline susceptibility, mechanistic biomarkers, real-world event signals, and treatment context to guide individualized monitoring intensity and intervention timing. That perspective matters because risk management is where the safety literature becomes clinically useful: it turns descriptive toxicology into decision support and gives the overall synthesis a forward-looking translational endpoint.

## 8 Clinical implications for practice

From a clinical perspective, effective management of drug-induced adverse events requires a shift from reactive recognition to proactive risk assessment and monitoring. Clinicians should consider toxicity risk as a dynamic interaction between drug mechanism, patient susceptibility, and treatment context, rather than as a fixed property of a given therapy.

Baseline risk stratification is essential prior to treatment initiation, as supported by both predictive modeling approaches and clinical studies identifying patient-specific risk factors for adverse events (Hu et al., 2018; Tian et al., 2023a). Factors such as age, comorbidities, organ function, prior treatment history, and concomitant medications should be systematically evaluated to identify patients at increased risk of adverse events. Particular attention should be given to populations with reduced physiological reserve, including older adults and patients with chronic organ dysfunction.

During treatment, dynamic monitoring plays a critical role in early detection of toxicity. Serial assessment of laboratory parameters (e.g., liver enzymes, renal function, cardiac biomarkers), together with symptom evaluation and, when appropriate, imaging or electrophysiological testing, can help identify early deviations from expected tolerance. Importantly, trends over time are often more informative than single measurements.

Mechanism-informed interpretation of toxicity is also crucial for clinical decision-making. Understanding whether an adverse event reflects on-target pharmacology, pathway-related effects, immune dysregulation, or metabolic stress can guide appropriate management strategies, including dose adjustment, temporary interruption, supportive therapy, or treatment discontinuation.

In addition, clinicians should integrate evidence from multiple sources, including clinical trials, real-world studies, and pharmacovigilance data, when evaluating safety signals. Rare or delayed toxicities may not be fully captured in pre-approval studies and often emerge in routine clinical practice (Li Z. et al., 2025).

Finally, individualized management strategies are essential. Not all adverse events require permanent discontinuation, and management should be tailored based on severity, reversibility, and the therapeutic benefit of continued treatment. Early recognition and timely intervention remain the most effective approaches to minimizing serious toxicity and improving overall treatment outcomes.

## 9 Predictive toxicology and future perspectives

The central challenge in contemporary drug safety is no longer limited to documenting adverse events after they occur. As pharmacotherapy becomes more targeted, biologically potent, and chronically administered across heterogeneous patient populations, the more important question is whether clinically meaningful toxicity can be anticipated before it becomes severe. Predictive toxicology therefore represents a bridge between mechanistic pharmacology, patient-level susceptibility, clinical monitoring, pharmacovigilance, and regulatory decision-making. Its value lies not in replacing clinical judgment, but in creating earlier and more structured opportunities to identify patients, drugs, combinations, and treatment contexts in which harm is more likely to emerge (U.S. Food and Drug Administration, 2009; International Council for Harmonisation, 2004; U.S. Food and Drug Administration, 2024).

Several mature examples already show how prediction can change clinical practice. Pharmacogenomic screening for HLA-B*57:01 before abacavir therapy is one of the clearest demonstrations that a severe immune-mediated adverse reaction can be reduced through pre-treatment genetic testing. Similar logic underlies HLA-guided carbamazepine prescribing to reduce the risk of severe cutaneous adverse reactions and TPMT/NUDT15-guided thiopurine dosing to reduce severe myelosuppression. These examples have already been incorporated into clinical guidelines, prescribing recommendations, or regulatory labeling, illustrating how predictive toxicology can move from evidence generation to clinical implementation. They are important because they move safety assessment from population-level caution to patient-specific risk stratification. They also show that predictive toxicology becomes clinically useful only when the marker is analytically reliable, clinically interpretable, linked to an actionable intervention, and accepted by prescribers within a practical workflow (Mallal et al., 2008; Leckband et al., 2013; Relling et al., 2019).

Biomarker-based prediction is also increasingly important for organ-specific toxicity. Drug-induced liver injury remains a major model for this problem because severe outcomes are uncommon, often idiosyncratic, and difficult to detect in premarketing trials of limited size. Regulatory guidance has emphasized the importance of systematic liver chemistry assessment, including aminotransferases, bilirubin, and clinical context, to distinguish transient enzyme elevations from patterns that suggest serious hepatocellular injury. In cardiotoxicity, cardio-oncology guidelines now integrate baseline cardiovascular risk, echocardiographic measures, global longitudinal strain, cardiac troponin, natriuretic peptides, cumulative dose, and treatment class to guide surveillance for anthracycline- and targeted therapy-related cardiac dysfunction. These approaches illustrate a broader principle: useful prediction rarely depends on a single marker. More often, it requires combining exposure, mechanism, patient susceptibility, baseline organ reserve, and longitudinal biomarker change (U.S. Food and Drug Administration, 2009; Lyon, 2022).

Mechanistic toxicology remains essential because many adverse events are not random clinical phenomena but predictable extensions of pharmacology in vulnerable tissues. EGFR inhibitor-associated rash reflects pathway inhibition in epithelial tissues; VEGF pathway inhibition produces vascular and renal toxicity through endothelial dysfunction; JAK inhibition alters host defense and inflammatory signaling; immune checkpoint blockade produces tissue-specific immune-related adverse events; SGLT2 inhibitors can promote ketoacidosis under susceptible metabolic conditions. These cases suggest that predictive toxicology should be organized around mechanistic motifs rather than isolated drug names alone. A mechanism-centered framework can help clinicians recognize why apparently different agents converge on similar toxicities, why certain patients are more vulnerable, and why monitoring should be tailored to both the drug and the host context (U.S. Food and Drug Administration, 2021; Salem et al., 2018; Lacouture, 2015; U.S. Food and Drug Administration, 2015; Li and Kroetz, 2018).

Real-world evidence and pharmacovigilance add a second predictive layer after approval. Randomized trials remain essential for estimating comparative safety under controlled conditions, but they may be underpowered for rare, delayed, or population-specific adverse events. Spontaneous reporting systems such as FAERS and VigiBase can identify early safety signals, as shown by pharmacovigilance analyses of immune checkpoint inhibitor-associated myocarditis and SGLT2 inhibitor-associated ketoacidosis. These systems are limited by reporting bias, missing denominators, incomplete clinical detail, and the inability to establish causality on their own. Nevertheless, they are valuable for signal generation, especially when combined with mechanistic plausibility and subsequent validation in cohorts, registries, claims data, or active surveillance networks. FDA Sentinel and similar distributed data systems represent an important evolution from passive signal detection toward more systematic, denominator-informed safety evaluation in routine care (U.S. Food and Drug Administration, 2024; Salem et al., 2018; Nguyen, 2022; U.S. Food and Drug Administration, 2015).

Artificial intelligence and computational toxicology may further strengthen this continuum, but their role should be framed carefully. Machine learning models can integrate chemical structure, omics signatures, electronic health records, spontaneous reports, imaging, laboratory trajectories, and clinical notes to prioritize potential safety signals or identify high-risk patients. Natural language processing may help detect immune-related adverse events, medication errors, or delayed toxicity patterns that are under-coded in structured data. In preclinical development, computational models can support prediction of drug-induced liver injury, QT prolongation, mitochondrial liability, carcinogenicity, and drug-drug interaction risk. However, these tools require external validation, transparent performance reporting, bias assessment, and clinical interpretability. A model that predicts a toxicity signal without explaining its uncertainty, population limits, or action threshold may increase noise rather than improve care (Bate and Luo, 2022).

Future safety frameworks should therefore move toward layered prediction rather than single-point prediction. At the preclinical stage, mechanistic assays, mitochondrial and transporter liabilities, toxicogenomic patterns, and exposure margins can identify early hazards. During clinical development, dose-response relationships, sentinel laboratory changes, cardiac and hepatic biomarkers, and adjudicated adverse-event phenotypes can refine risk. After approval, spontaneous reports, active surveillance, real-world cohorts, and digital health data can detect rare or delayed events. At the bedside, pharmacogenomics, organ function, comorbidities, frailty, drug-drug interactions, prior toxicity history, and patient preferences must be translated into monitoring and management decisions. The ultimate goal is not merely to predict that an adverse event could occur, but to define what should be done differently because of that prediction (U.S. Food and Drug Administration, 2009; International Council for Harmonisation, 2004; U.S. Food and Drug Administration, 2024; Lyon, 2022).

Several gaps remain. First, many proposed biomarkers and computational models have not been validated across ancestry groups, age ranges, comorbidity burdens, and care settings. Second, adverse-event definitions are often inconsistent across trials, spontaneous reports, and real-world datasets, making cross-study comparison difficult. Third, mechanistic plausibility and statistical signal detection are not always aligned; a strong disproportionality signal may lack biological explanation, whereas a compelling mechanism may not yet have robust clinical evidence. Fourth, predictive tools can worsen inequity if they are trained on incomplete or non-representative datasets. Finally, risk prediction must be linked to feasible clinical action. Screening tests, monitoring schedules, risk scores, and AI alerts are useful only if they improve decisions without creating excessive burden, alarm fatigue, or inappropriate withholding of effective therapy (Bate and Luo, 2022).

In this context, predictive toxicology should be viewed as a practical extension of modern pharmacotherapy rather than a separate technical discipline. The field is moving from descriptive adverse-event catalogues toward integrated risk models that connect drug mechanism, patient susceptibility, biomarkers, evidence hierarchy, and regulatory action. For clinicians, this shift supports earlier recognition, individualized monitoring, and more transparent benefit-risk discussion. For researchers, it highlights the need for validated biomarkers, interoperable safety datasets, and mechanistically interpretable models. For regulators and health systems, it reinforces the importance of pharmacovigilance planning, active surveillance, and timely risk communication. The most clinically useful safety science will be the kind that turns prediction into prevention, mitigation, or better-informed treatment selection (U.S. Food and Drug Administration, 2009; International Council for Harmonisation, 2004; U.S. Food and Drug Administration, 2024).

## 10 Perspective and conclusion

Drug-induced adverse events are no longer adequately captured by lists of organ-specific complications. Across modern pharmacotherapy, toxicity increasingly reflects the redistribution of pharmacologic effects across interconnected biological systems, where the same pathways that mediate therapeutic benefit can produce injury under different conditions of exposure, tissue context, and patient susceptibility (Jayathilaka et al., 2024; Turkington et al., 2025; Hushmandi et al., 2026).

This shift also highlights a broader limitation in how safety evidence is traditionally interpreted. No single layer of evidence, whether randomized trials, meta-analyses, or real-world data, can fully define risk. Instead, each captures a different dimension of safety, and their value lies in how they are integrated. Mechanistic insight provides biological plausibility, pooled analyses clarify comparative risk, and observational data reveal patterns that only emerge in routine clinical use. Bringing these elements together is essential for producing safety assessments that are both interpretable and clinically useful (Jiao et al., 2024; Niu et al., 2025; Rathod et al., 2026).

Building on this integrated view, an important transition is underway from retrospective detection of toxicity to more anticipatory approaches to risk management. Early biomarker changes, dynamic monitoring strategies, and structured decision frameworks are beginning to support earlier intervention and more precise adjustment of therapy (Fu et al., 2020; Cao et al., 2026; Shojaei et al., 2026). This is particularly relevant in settings where treatments are prolonged, combined, or applied to heterogeneous patient populations, all of which increase the likelihood that toxicity will evolve over time rather than appear as a single discrete event.

Looking forward, further progress will depend less on the accumulation of additional safety reports and more on improving how existing evidence is connected and applied. Predictive models that combine baseline susceptibility, exposure characteristics, mechanistic understanding, and real-world signals offer a pathway toward more individualized risk assessment. At the same time, advances in pharmacovigilance informatics and artificial intelligence are likely to accelerate signal detection, but their clinical value will depend on integration with context-aware interpretation rather than isolated pattern recognition (Bate and Luo, 2022; Chen S. et al., 2025; He et al., 2025).

From a clinical perspective, these developments underscore the need to translate complex safety evidence into actionable decision-making frameworks. Clinicians should integrate mechanistic insight, patient-specific risk factors, and evolving real-world safety signals when selecting therapies, planning monitoring strategies, and responding to emerging adverse events.

Taken together, pharmacotherapy safety is best understood as a dynamic and multi-layered system linking biology, evidence, and clinical decision-making. Within this framework, safety is not a secondary constraint on therapeutic innovation, but a central component that determines how treatments can be used effectively, adapted to patient context, and sustained in real-world practice. From a clinical perspective, integrating these elements into routine decision-making is essential for improving patient safety and optimizing therapeutic outcomes.
